# Corolla Abscission Triggered by Nectar Robbers Positively Affects Reproduction by Enhancing Self-Pollination in *Symphytum officinale* (Boraginaceae)

**DOI:** 10.3390/biology10090903

**Published:** 2021-09-13

**Authors:** Qin-Zheng Hou, Nurbiye Ehmet, Da-Wei Chen, Tai-Hong Wang, Yi-Fan Xu, Jing Ma, Kun Sun

**Affiliations:** College of Life Sciences, Northwest Normal University, Lanzhou 730070, China; hou_qzh@nwnu.edu.cn (Q.-Z.H.); nuerbiya123456@163.com (N.E.); gansudaweichen@126.com (D.-W.C.); wang_tho@163.com (T.-H.W.); xuyifan0825@126.com (Y.-F.X.); mjing1121@163.com (J.M.)

**Keywords:** *Symphytum officinale*, nectar robber, corolla abscission, delayed self-pollination, reproductive assurance

## Abstract

**Simple Summary:**

Nectar robbers affect plant fitness in different degrees and in different ways, potentially constituting an important part of pollination interactions. While the negative effects of nectar robbing on plant reproductive success have been widely reported, the positive effects are quite unclear. Hence, our study was designed to assess the effects of nectar robbers on reproductive success of *Symphytum officinale*. This will help in understanding the evolutionary significance of mutualistic relationships between plants and their visitors.

**Abstract:**

Nectar robbers, which affect plant fitness (directly or indirectly) in different degrees and in different ways, potentially constitute a significant part of mutualistic relationships. While the negative effects of nectar robbing on plant reproductive success have been widely reported, the positive effects remain unknown. The target of our study was to evaluate the effects of nectar robbers on the reproductive success of *Symphytum officinale* (Boraginaceae). We observed the behavior, species and times of visitors in the field, and we assessed the effect of nectar robbers on corolla abscission rate and time. To test the fitness of corolla abscission, we detected the changes in stigma receptivity, pollen viability, pollen amount and appendage opening size along with the time of flower blossom. The flowering dynamics and floral structure were observed to reveal the mechanism of self-pollination. Finally, pollen deposition seed set rate and fruit set rate were determined to estimate the effect of nectar robbers on reproduction success. We observed 14 species of visitors and 2539 visits in 50 h of observation; 91.7% of them were nectar robbers. The pressure and nectar removal of nectar robbers significantly promoted corolla abscission during a period when pollen grains are viable and the stigma is receptive. In addition, corolla abscission significantly increased the pollen deposition and seed setting rate. Our results demonstrate that nectar robbing contributes to enhancing seed production and positively and indirectly impacts the reproductive success of *S. officinale*. This mechanism involved the movement of anthers and indirect participation by nectar robbers, which was rarely investigated. Considering the multiple consequences of nectar robbing, understanding the impact of nectar robbers on plant reproduction is essential to comprehend the evolutionary importance of relationships between plants and their visitors.

## 1. Introduction

Mutualistic interaction between plants and pollinators is an important part of ecosystem function. The mutualistic interaction between plants and pollinators evolved from adaptive speciation interaction by animals and plants [1,2,3]. Plants and pollinators generally have a mutualistic relationship where pollinators benefit from plant resources, such as pollen or nectar, while plants receive outcrossing pollen to fertilize ovules and ensure successful reproduction [4,5,6]. However, sometimes plants and pollinators may experience conflict because they have different interests. It is in the best interest of the pollinator to maximize the collection of the floral reward for food [7], relative to handling costs, while it is in the best interest of the plant to maximize pollen receipt and transfer [8]. For instance, nectar robbers steal nectar without contributing to pollination [2].

Nectar robbers are flower visitors who, due to competition with other flower visitors or morphological trait mismatch with the visited flower, steal floral nectar [9]. Nectar robbers extract nectar by piercing the flower, whereas nectar thieves visit the flowers as pollinators but with little or no effect on pollination [10]. According to previous studies, nectar robbers are extensively distributed geographically and are represented by a variety of species [11], including both insects and birds. Nectar robbing is divided into two categories: primary nectar robbing (PNR), in which a slit is created by chewing or slicing the flower’s corolla to obtain nectar [12,13], and secondary nectar robbing (SNR), in which nectar is obtained by a slit previously made by a primary robber [14]. Secondary robbers include some existing legitimate visitors and flower visitors who cannot obtain nectar through the legitimate flower entrance [14,15]. Due to the mismatch between visitor tongue and flower shape, short-tongued visitors are likely to be the primary nectar robbers [16]. The behavior of nectar robbers may have significant evolutionary and ecological consequences on the plant populations that they target [2].

Many studies have been carried out on the ecology of nectar robbing in plant–pollinator mutualisms, particularly from the plant perspective [17]. Nectar robbing has an obvious negative connotation with adverse effects on the fitness of plants [18]. For instance, nectar robbers negatively affect plant fitness by decreasing the visitation rate of the legitimate pollinators [19,20], destroying floral structures [21,22] or reducing the availability of nectar volume [19]. This has been deemed to reduce the attractiveness to pollinators and hence influences the plant’s reproductive success [23,24]. However, recent meta-analyses and reviews indicate that the effects of nectar robbing might be neutral, when antirobbery did not increase the plant reproductive success [25,26]. Even positive effects have been reported on plant reproductive success [12,27]. Therefore, just as the influence of herbivores changes from negative to neutral and then to positive, so does the influence of nectar robbers [2]. Numerous pathways and mechanisms can result in the direct and indirect outcomes of nectar robbing on plant reproduction, many of which are similar to those of herbivores that affect plant fitness [28]. Although the phenomenon of nectar robbing is of common occurrence [9], positive effects have only rarely been reported in previous studies [29,30]. If nectar robbers are detrimental to plant fitness, why has this asymmetric relationship existed for a long time? We believe that there is a mutual interaction between nectar robbers and plants resulting from coevolution; the current research on this complex ecological relationship is partial and limited, and the evidence on the key attributes that resulted in these mutual interaction frameworks have only recently begun to emerge [31,32].

In the present study, we investigated the effect of nectar robbing on plant reproductive fitness in *Symphytum officinale* L. *S. officinale* is a perennial flowering plant in the family Boraginaceae and is open mainly from May to October. Preliminary field observations indicated that most visitors of *S. officinale* are nectar robbers with a high visiting rate. So what is the adaptive fitness benefit of this large number of nectar robbers to *S. officinale*? Through our observations, we found that the nectar robbers hold the flower tightly and cause a downward drag pressure on the flower when visiting flowers, and almost all the nectar was removed after one visit. Therefore, we speculate that these two behaviors (nectar removal and pressure) of nectar robbers may promote the corolla abscission. In addition, the flower of *S. officinale* has a peculiar structure in which throat appendages tightly gather anthers parallel adnate to the style below the stigma). This structure ensures that the anthers can slip along the style at corolla abscission and the anthers are dragged across the stigma by the moving corolla. Previous studies have reported the case of self-pollination promoted by corolla abscission in *Mimulus guttatus* [33,34] and *Incarvillea sinensis* var. *sinensis* [35]. It was therefore hypothesized that the nectar robbers might play a significant role in the corolla abscission and leading to anther stigma contact, and they may realize delayed self-pollination, that is, pollen–stigma contacts at the end of anthesis. Therefore, the delayed self-pollination caused by nectar robbers might contribute to sexual reproductive fitness positively. If so, it will be a new discovery in plant–pollinator interactions. The aim of this research is to confirm this prediction for *S. officinale* by addressing the following questions: (1) Do nectar robbers trigger corolla abscission? (2) Does corolla abscission facilitate delayed self-pollination, and how? (3) If so, to what degree do the nectar robbers contribute to seed and fruit production?

## 2. Materials and Methods

### 2.1. Study Sites

*S. officinale* is a perennial flowering plant in the family Boraginaceae. Each plant supports a variable number of inflorescences, up to 25 tubular flowers, with approximately 20–25 open at the same time, which are open mainly from May to October.

The experiment was conducted from May to October 2020 in Lanzhou Shifogou National Forest park (long. 103°50′50″, lat. 35°55′00″, alt. 1990), in Lanzhou, China. The average annual temperature of the site is 7.8 °C, and the average annual rainfall is 380 mm.

### 2.2. Pollinator Observations

To quantify and identify visitors on *S. officinale* flowers during anthesis, we conducted surveys in the field between 08:00 a.m. and 18:00 p.m. for five days. During the surveys, we recorded the foraging behavior and the visitation frequency of visitors. We randomly selected 6 individual plants and monitored 10 fresh flowers per individual per day. We observed each individual plant for 10 min per hour (100 min per day), comprising an overall 50 h sampling, 5 days and 30 sampled plants. Insect specimens were collected in specimen boxes for later identification.

We sorted out the behavior variants of visitors by using the classification based on the work of Inouye [36]. First, we classified visits as legitimate visits (LVs) when visitors gather nectar through the corolla entrance, contacting the stigma and the anthers, and illegitimate visits (IVs) when visitors gather nectar without touching the stigma or the anthers. Then, we tested 2 IV types: PNR and SNR.

### 2.3. Contribution of Nectar Robbers to Corolla Abscission

**Figure 1:**
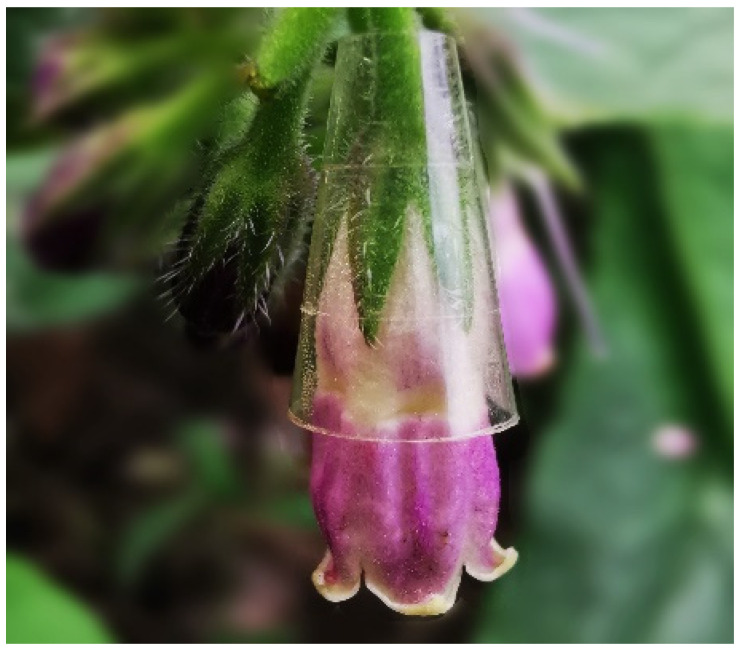
Flower of *Symphytum officinale* fitted at the base of corolla by a collar made of adhesive tape to prevent corolla from being pierced by nectar robbers.

We used six treatments (*n* = 30 flowers respectively) to estimate the contribution of corolla abscission to plant fitness: (1) bagged, in which flowers were bagged before blossom, avoiding the influence of visitors; (2) natural condition, without any treatments; (3) antirobbing, in which a collar was fit on the base of the corolla tubes to prevent robbing (Figure 1) (The collar consisted of adhesive tape that could prevent the corolla tube from being pierced. We observed the unsuccessful visits of nectar robbers, as well as usual pollinator activity, during the experiment to ensure that influences on pollinator visitation due to the collar were insignificant.); (4) nectar removal, in which flowers were bagged and nectar was removed with a graduated microsyringe (25 μL Hamilton) every two hours until flower abscission; (5) artificial pressure, in which insect pressure (average weight of 12 species of nectar robbers) on flowers by was simulated by a digital push–pull gauge force gauge (KTE HF-5, 0.001 N–5 N); and (6) artificial pollination, in which flowers were bagged and manually pollinated with pollen collected from other plants 1000 m away. After each treatment, the flower condition (corolla abscission or not) was observed every 4 h until corolla abscission. The normality of data was tested using 1-K-S, and then one-way ANOVAs (with Tukey’s multiple contrasts) were used to test the difference in time of corolla abscission between the different treatments.

We randomly tagged 300 buds on different individuals and collected 30 flowers at every 12 h interval after blossom (until the corolla fell off) to test for changes in stigma receptivity, pollen viability, pollen amount and appendage opening size along with the time of flower blossom. The pollen viability was detected as the percentage of germinated pollen grains by pollen germination experiment, and the stigma receptivity was tested by the benzidine/H_2_O_2_ method [37] and activity of POD, SOD and CAT. The activities of POD and CAT were determined by guaiacol colorimetry, the activity of SOD was determined by NBT-illumination method.

### 2.4. Floral Biology

Thirty buds were randomly tagged from 10 individual plants, and we recorded the phenological development of each flower. The time of flower opening and abscission, anther and stigma presentation, floral traits and the movements of floral structures were recorded during a 2 h period between 9:00 a.m. and 6:00 p.m.

To observe the process of anther movement at corolla abscission, half of the corolla was cut off longitudinally and dragged along the style with tweezers, simulating the process of corolla abscission. Then, the movement of floral structures in the process of corolla abscission was photographed.

To determine the nectar-secreting pattern of *S. officinale*, we measured nectar concentration and volume [37] in 30 bagged flowers every four hours until flowers dropped. We removed the nectar and measured its volume with a graduated microsyringe (25 μL Hamilton), and we estimated the sugar concentration with a refractometer (0–90% Brix; mod. RT-280, Atago).

### 2.5. Adaptive Significance of Corolla Abscission

To estimate the contribution of corolla abscission to pollen deposition and seed and fruit set rate, eight treatments (*n* = 30 for each treatment) were conducted: (1) natural pollination; (2) bagged, the same method as mentioned in Section 2.3; (3) antirobbing, the same method as mentioned in Section 2.3; (4) emasculation, in which the anthers were removed before dehiscence and pollination occurred naturally; (5) appendage removal, in which the appendages were removed upon anthesis initiation; (6) hand cross-pollination, in which flowers were emasculated and manually pollinated with pollen grains from other individuals ≥ 1000 m away; (7) hand self-pollination, in which flowers were manually pollinated by own pollen grains; and (8) emasculated and bagged. The stigmas (30 flowers each treatment) were collected after corolla abscission to detect the pollen deposition, measured by microscopic examinations after staining with lactophenol cotton blue. The seed set rate and fruit set rate were detected after 30 days of flower exposure. The normality of data was tested using 1-K-S, and then one-way ANOVAs (with Tukey’s multiple contrasts) were used to test the difference in pollen deposition, seed set rate and fruit set rate among the different treatments.

## 3. Results

### 3.1. Visitor Observations

**Figure 2:**
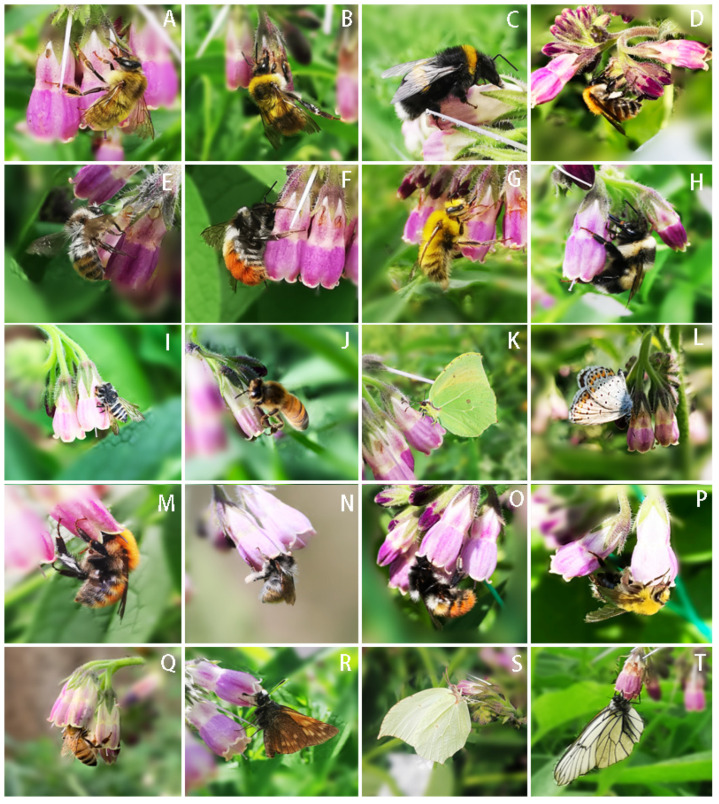
Visitors of *S. officinale*. (**A**–**L**): Illegitimately visit flowers (IV), gather nectar on base of corolla. M–T: Legitimately visit flowers (LV), gather nectar through the corolla entrance. (**A**) *Bombus picipes*, (**B**) *Bombus longipennis*, (**C**) *Bombus lucorum*, (**D**,**M**) *Bombus hedini*, (**E**,**N**) *Bombus ladakhensis*, (**F**,**O**) *Bombus kashmirensis*, (**G**,**P**) *Bombus laesus*, (**H**) *Bombus lantschouensis*, (**I**) *Megachile rotundata*, (**J**,**Q**) *Apis mellifera*, (**K**,**S**) *Gonepteryx mahaguru*, (**L**) *Everes argiades*, (**R**) *Ochlodes subhyaline*, (**T**) *Aporia crataegi*.

We observed a total of 2539 individual floral visits and 14 insect species in 50 h of observation on *S. officinale* flowers (Table 1). Visitors exhibited a diversity of feeding behaviors, including LVs and IVs (PNR and SNR). Eight species legitimately visited *S. officinale* flowers in our sampling, including four species of bumblebee, one species of honeybee and three species of butterflies. Twelve species illegitimately visited *S. officinale* flowers, including nine bumblebees, two honeybees and one species of butterflies. The number of visits varied according to legitimacy; LVs accounted for 8.23% and IVs accounted for 91.76% (PNR 7.20%, SNR 84.56%). The number of visits and visit behavior of each species are shown in Table 1, and photographs of the visitors are shown in Figure 2.

Among the eight species of insects legitimately visiting, only three species of bumblebees pollinated effectively, *Bombus hedini*, *B. ladakhensis* and *B. kashmirensis*. The butterflies collected nectar through the flower entrance but did not touch the stigma and anthers; illegitimate visitors gather nectar illegally from the base of the corolla, also without touching the stigma and anthers, and hence do not contribute to the pollination (Table 1)**.**

### 3.2. Contribution of Nectar Robbers to Corolla Abscission

**Figure 3:**
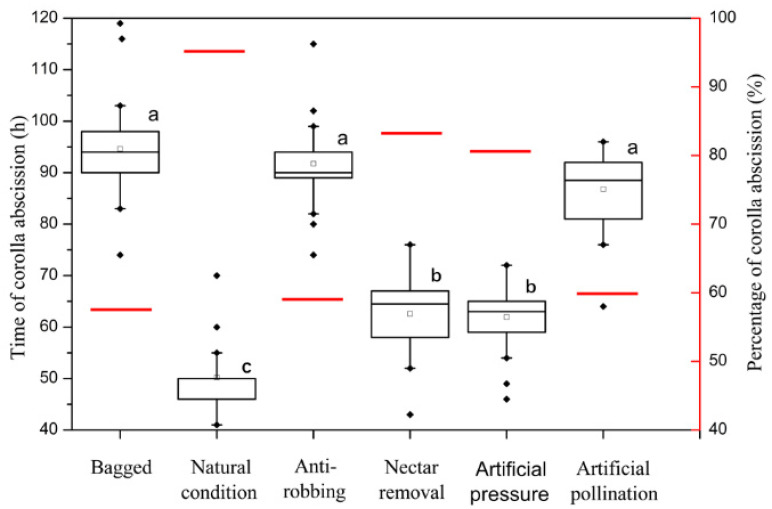
Time and proportion of corolla abscission under different treatments. Boxplots indicate the time of corolla abscission, showing medians, quartiles, interquartile ranges and outliers. The red line indicates the proportion of corolla abscission. Different letters on items indicate significant difference at the 0.05 level.

The time and proportion of corolla abscission of bagged flowers were 94.6 ± 6.1 h and 56.6%, respectively. Compared with bagged flowers, the antirobbing (92.1 ± 7.6 h, 65%) and artificial pollination (89.4 ± 6.2 h, 60%) were not significantly different in time and proportion of corolla abscission. However, the artificial pressure (63.2 ± 4.8 h, 80%), nectar removal (64.3 ± 5.2 h, 83.3%) and natural condition (49.8 ± 5.1 h, 96.7%) flowers exhibited a significant reduction in time of corolla abscission and a significant increase in proportion of abscised corollas (Figure 3). This indicated that the pressure of insects and nectar removal had a significant influence on corolla abscission, showing that the nectar robbers significantly promoted the corolla abscission.

**Figure 4:**
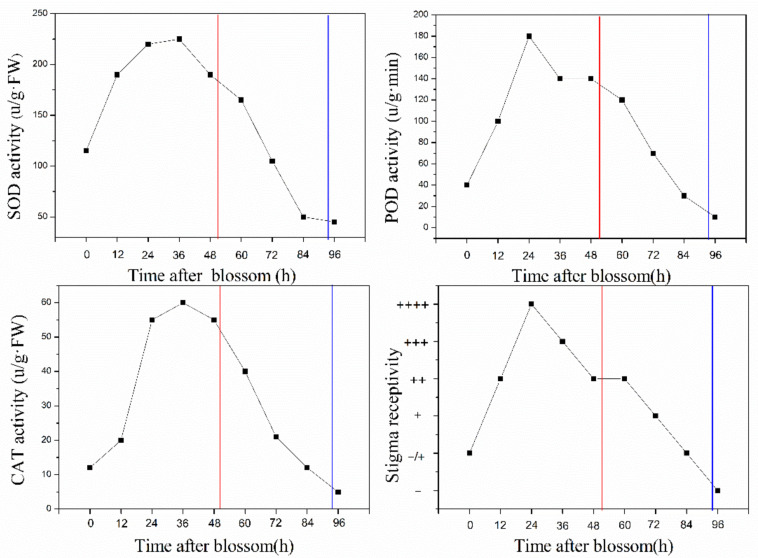
The dynamics of stigma receptivity and activity of POD, SOD and CAT over time after blossom. “−” means the stigma is not receptive; “−/+” means that some of the stigmas are receptive; “+” means the stigma is receptive, and more “+” symbols mean the stigma is strongly receptive. The red vertical lines refer to time of natural corolla abscission, and the blue vertical lines refer to time of corolla abscission after anti-robbing.

**Figure 5:**
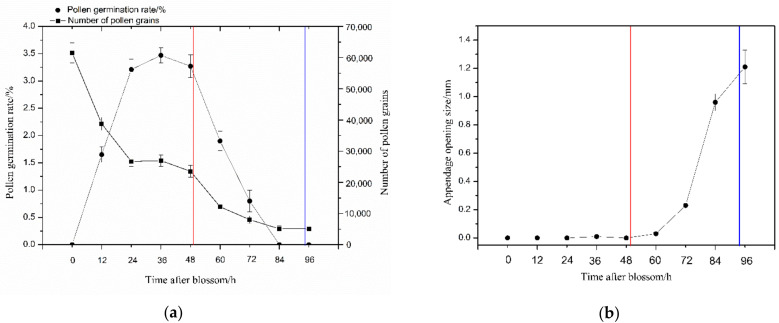
(**a**) The dynamics of the pollen germination rate and number of pollen grains over time after blossom; (**b**) appendage opening size over time after blossom. The red vertical lines refer to time of natural corolla abscission, and the blue vertical lines refer to time of corolla abscission after antirobbing.

The stigma receptivity was lower at the beginning of flowering (0 h), peaked between 24 and 36 h after blossom and remained receptive (++) at the corolla abscission naturally (49.8 ± 5.1 h). Subsequently, the stigma receptivity decreased gradually and was lower at 72 h, and no peroxidase activity was detected at 96 h. Correspondingly, the activities of SOD, POD and CAT changed in the same way (Figure 4). Determination of pollen germination rate shows that it was 1.2% when the anthers dehisced, peaked at 3.5% at about 36 h and remained higher (3.3%) when the corollas abscised (49.8 ± 5.1 h), but no germinated pollen grains were detected at 96 h (Figure 5a). The pollen amount available was decreased with flowering time. *S. officinale* flowers have an average of 61,505 ± 4716 pollen grains. A mean of 38.26 ± 3.7% pollen grains per anther remained when the corollas abscised naturally (49.8 ± 5.1 h), 13.05 ± 1.8% remained at 72 h and only 8.27 ± 2.4% remained at 96 h (Figure 5a). In addition, the dynamics of the appendage opening size showed that the appendages did not open within 48 h after blossom; it was still 0 mm when the corollas abscised (49.8 ± 5.1 h). Subsequently, the appendage opening size increased gradually, reaching 1.21 + 0.12 mm at 96 h (Figure 5b).

### 3.3. Floral Biology

The stigma was already receptive at 0 h, the anthers dehisced 6 ± 0.76 h after flower opening and the unwilted corollas slipped forwards and abscised 49.8 ± 5.1 h after flower opening.

**Figure 6:**
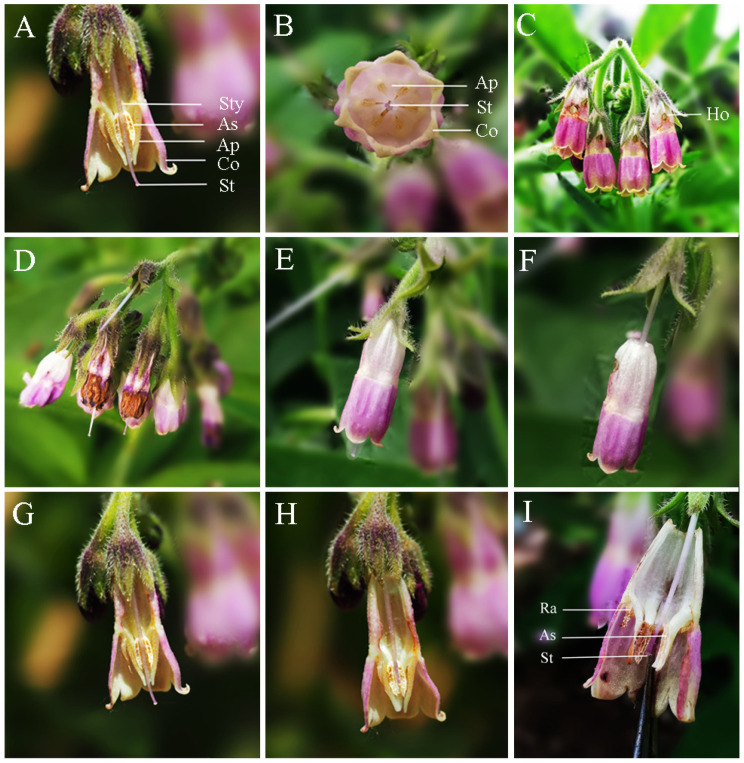
The floral traits and process of corolla abscission. (**A**–**C**) The morphological characteristics for *S. officinale*, comprising stamens and pistil in the opening flower. St, stigma; Co, corolla; Ap, appendages; As, anther sac; Sty, style; Ho, holes made by nectar robbers. (**D**) The wilted flower in the insect-excluding bags. (**E**,**F**) Corolla abscission and slipping along the style. (**G**,**H**) Anther movement and the process of self-pollination upon corolla abscission. (**I**) The process of corolla abscission after removal of appendages: Ra, residues of appendages; As, anther sac; St, style.

Inflorescences of *S. officinale* have many flowers with tubular corolla (Figure 6C), and the flowers have five throat appendages, five pistils and one stamen inside the corolla; the pistil and stamen are isolated by throat appendages (Figure 6A,B). Throat appendages tightly gather anthers parallel adnate to the style under the stigma (Figure 6A). This structure ensures that the anthers can slip along the style at corolla abscission.

When the corolla abscised, the anthers were dragged by the corolla and slipped along the style under the gatherings imposed by the appendages. Finally, the anthers were dragged across the stigma by the moving corolla. The anther dehiscence faces contacted the surfaces of stigma and brushed pollen onto it after the anthers passed the stigma (Figure 6E–H). When the appendages were removed before flowering, the anthers scattered from the style, preventing the anthers from touching the surfaces of stigma when the corolla abscised (Figure 6I).

**Figure 7:**
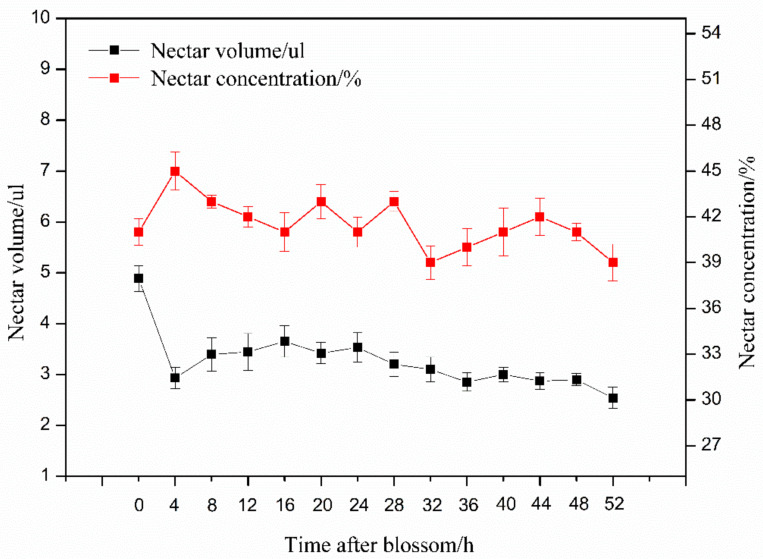
The volume and concentration of nectar every 4 h after blossom. The red line refers to nectar concentration and the black line refers to nectar volume.

*S. officinale* flowers continuously secreted nectar until corolla abscission, and flowers accumulated approximately 15.1% (4.89 ± 0.25 μL) of total nectar volume before opening (45.71 ± 5.22 μL). Flowers accumulated a nectar volume similar to that secreted before the flower opened in an eight-hour interval. This indicated that the robbed flowers were continuously replenished with nectar after each robbing, and no differences in sugar concentration and nectar volume were found between different flowering times (Figure 7).

### 3.4. Adaptive Significance of Corolla Abscission

**Figure 8:**
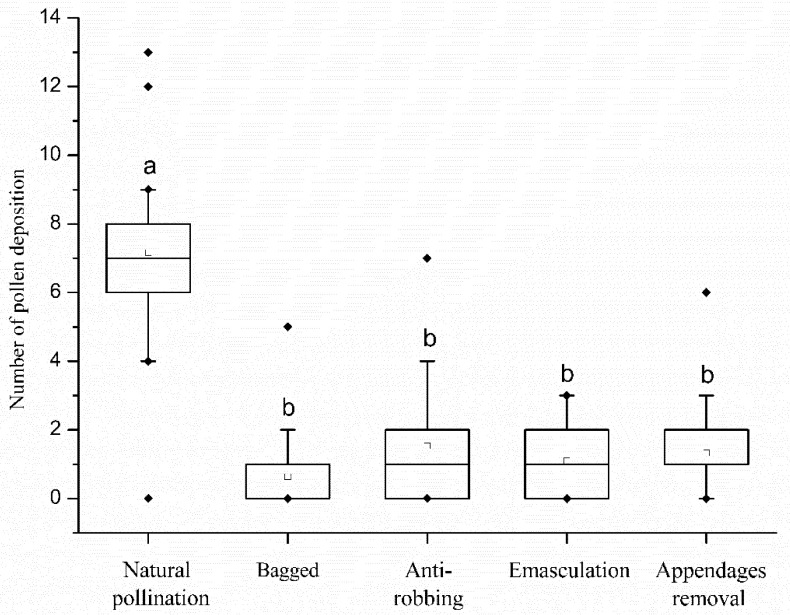
Comparison of pollen deposition number between different treatments. Boxplots show medians, quartiles, interquartile ranges and outliers. Different letters on items indicate significant difference at the 0.05 level.

Under natural conditions, we detected 7.1 ± 0.89 pollen grain depositions on the stigma (Figure 8), and the fruit set rate and seed set rate were 5 ± 21.8% and 1.25 ± 6.9% respectively (Table 2). The bagged treatment showed a lower pollen deposition number and did not develop into fruit, indicating that the *S. officinale* has no ability of spontaneous self-pollination. The pollen deposition and seed setting rate decreased significantly after the antirobbing, emasculation and removal of appendages (Figure 8; Table 2). In addition, the fruit and seed sets followed by manual self-pollination and manual cross-pollination showed no significant difference, showing that *S. officinale* is self-compatible. The bagged flowers with emasculation did not bear seeds, indicating that this species is not capable of apogamy. These results showed that the pollinators had low pollination efficiency; therefore, the *S. officinale* mainly propagates by self-pollination. Nectar robbers played important roles in the reproduction of *S. officinale*.

## 4. Discussion

Long corolla tubes are a classical example of nectar barriers because they prevent undesired visitors from consuming the reward intended for their long-tongued, most effective pollinators [38]. The flower attractiveness and nectar rewards may increase as the investment in resource barriers increases [39]. However, long corolla tubes also increase the rate of robbing by visitors that pierce holes in the base of the corolla to obtain nectar without pollination. Our results showed that the long corolla of *S. officinale* secretes a high volume of nectar (Figure 7), and this attracted a large number of nectar robbers to visit (Table 1). We found that all the recorded robbers held the flower tightly when visiting flowers and stole nectar from the base of the corolla (Figure 2), thereby causing two effects on the flowers, namely pressure and removing nectar. The pressure caused a downward drag on the flower, and the removal of nectar prevented the base of the corolla from sticking on the ovary. The combination of these two effects increased the time and rate of corolla abscission (Figure 3). Therefore, our results support the point that the behavior of nectar robbers promotes corolla abscission [11,40].

Previous studies have reported that the corolla abscission facilitated delayed self-pollination through special flower structures in *Mimulus guttatus* [33,34] and *Incarvillea sinensis* var. *sinensis* [35]. The present work demonstrates that the throat appendages tightly gather anthers parallel adnate to the style under the stigma. This structure ensures that the anthers can slip along the style and brush pollen onto the stigma at the corolla abscission (Figure 6E–H), and it significantly increases pollen deposition on stigma (Figure 8). Therefore, it can be recognized as a specialized structure for self-pollination promoted by anther-dragging corolla movement. In addition, the stigmas of *S. officinale* are receptive (Figure 4), and a higher number of viable pollen grains remain (Figure 5a) when the corolla abscises; thus, self-pollination is achieved. On the contrary, excluding insects or nectar robbers significantly prolonged the time of corolla abscission and reduced the proportion of corolla abscission (Figure 3). This resulted in the loss of stigma and pollen grain vitality (Figure 4 and Figure 5a) and lead to a small amount of pollen remaining (Figure 5a) when the corolla abscised, leading to the impossibility of self-pollination. This indicated that the nectar robbers promoted corolla abscission and thereby realized self-pollination.

The present artificial-pollination experiments indicated that *S. officinale* is self-compatible. Therefore, the increased numbers of pollen grains deposited on the stigma resulted in an increase in seed set rate through self-pollination within flowers. The fruit and seed set rates of flowers prevented from abscising were significantly lower than those of flowers that naturally abscised. This indicated that the corolla abscission significantly enhanced the seed set rate by self-pollination. However, the cross-pollination occurred before the corolla fell off and only deposited 1.1 ± 0.84 pollen grains on the stigma. Considering the four ovules in one flower and low pollen viability, the pollinator cross-pollination contributed slightly to seed production. In this species, outcrossing results only from pollinator visitation before corolla abscission, and inbreeding is caused by nectar-robber-dragged corolla abscission. Therefore, there are no opportunities for outcrossing after corolla abscission. According to the definitions of Lloyd [41], this mechanism of self-pollination is regarded as delayed self-pollination. In the present case, nectar robbers play an important role in the mechanism of self-pollination driven by corolla abscission, and the advantage of this mechanism appears to outweigh the possible negative effects such as pollen discounting by geitonogamy. We, therefore, consider that the nectar robbers play an indirectly positive effect on the fitness of *S. officinale*. This is in contrast to some other studies where nectar robbing results in negative effects due to pollinator limitation [29,30,31].

According to our results, we believe that nectar robbers are a key part of mutualistic plant–insect interactions in some systems and that a combination of ingenious mechanisms leads plants to compensate for the resource investment in nectar consumed by robbers. Our research offers further evidence that plant–insect interactions are complex and may consist of multiple interaction mechanisms occurring simultaneously [42,43,44,45]. It has been some questioned how mutualisms can sustain over an evolutionary time scale under such exploitation. In the face of such problems, perhaps we need to rethink the definition of nectar robbery being completely exploitative. The phenomenon of nectar robbing may not only have an influence on community-level interactions and plant population growth [46] but also impact the stability of the plant–pollinator interaction network. In addition, considering that the benefits and costs are not static in a plant–pollinator mutualistic interaction and may vary with species composition in a community or seasons [47], future research needs to explore the long-term effects of nectar robbery on plants’ reproductive fitness.

## Figures and Tables

**Table 1:** Number of legitimate visits (LVs) and illegitimate visits (IVs: PNR = primary nectar robbing; SNR = secondary nectar robbing) per visitor on *S. officinale* and pollination efficiency.

Group	Species	Number of Visits				Pollination Efficiency		
		LVs	PNR	SNR	Total	LVs	PNR	SNR
Bumblebees	*Bombus hedini* Bischoff, 1936	74	-	83	157	3.5 ± 0.70	-	-
	*Bombus picipes* Richards, 1934	-	-	104	104	-	-	
	*Bombus ladakhensis* Richards, 1928	22	-	205	227	4.25 ± 1.5	-	
	*Bombus longipennis* Friese, 1918		-	18	18	-	-	-
	*Bombus lucorum* Linnaeus, 1761	-	104	344	448	-	-	-
	*Bombus kashmirensis* Friese, 1909	39	-	995	1034	1 ± 0.21	-	-
	*Bombus laesus* Morawitz, 1875	27	-	222	249	-	-	-
	*Bombus lantschouensis* Vogt, 1908	-	79	116	195	-	-	-
Honeybees	*Apis mellifera* Linnaeus, 1758	2	-	23	25	-	-	-
	*Megachile rotundata* Fabricius, 1793	-	-	16	16	-	-	-
Butterflies	*Aporia crataegi* Linnaeus, 1758	11	-	-	11	-	-	
	*Everes argiades* Pallas, 1771	-	-	9	9	-	-	-
	*Gonepteryx mahaguru* Gistl, 1857	14	-	12	26	-	-	-
	*Ochlodes subhyalina* Bremer and Grey, 1853	20	-	-	20	-	-	-
	Total	209	183	2147	2539	-	-	-

**Table 2:** Comparison of seed set and fruit set between different treatments.

Treatments	Natural Pollination	Bagged	Antirobbing	Emasculation	Appendage Removal	Artificial Cross-Pollination	Artificial Self-Pollination	Bagged and Emasculation
Seed set rate/%	1.50 ± 2.05 ^b^	0 ^c^	0.08 ± 0.15 ^c^	0.16 ± 0.3 ^c^	0.08 ± 0.21 ^c^	2.41 ± 5.15 ^a^	1.80 ± 2.45 ^ab^	0 ^c^
Fruit set rate/%	5 ± 12.5 ^b^	0 ^c^	0.33 ± 1.5 ^c^	0.67 ± 2.05 ^c^	0.33 ± 1.5 ^c^	9 ± 20.8 ^a^	7 ± 18.5 ^ab^	0 ^c^

Different superscript letters within the same row indicate significant difference at *p* < 0. 05.

## Data Availability

The data presented in this study are available on request from the corresponding author.
